# NAILS: Normalized Artificial Intelligence Labeling Sensor for Self-Care Health

**DOI:** 10.3390/s24247997

**Published:** 2024-12-14

**Authors:** Livio Tenze, Enrique Canessa

**Affiliations:** The Abdus Salam International Centre for Theoretical Physics (ICTP), 34151 Trieste, Italy

**Keywords:** region-based image segmentation, machine learning algorithm, fingernail color analysis, early disease detection

## Abstract

Visual examination of nails can reflect human health status. Diseases such as nutritive imbalances and skin diseases can be identified by looking at the colors around the plate part of the nails. We present the AI-based NAILS method to detect fingernails through segmentation and labeling. The NAILS leverages a pre-trained Convolutional Neural Network model to segment and label fingernail regions from fingernail images, normalizing RGB values to monitor tiny color changes via a GUI and the use of an HD webcam in real time. The use of normalized RGB values combined with AI-based segmentation for real-time health monitoring is novel and innovative. The NAILS algorithm could be used to self-extract and archive primary signs of diseases in humans, especially in rural areas or when other testing may be not available.

## 1. Introduction

The examination of the nails in the limbs of a person plays an important role in medicine. In human beings, nails are the farthest part of the body from the heart and are the last part to receive oxygen. Hence, an attentive observation of nails can be one initial method to check the physiological condition of the human body. Diseases can be identified by looking particularly at the lunula and plate parts of the nail. Depending upon their shape, texture, and color, these analyses can predict early symptoms of diseases and can be indicators of, e.g., nutritive imbalances, skin diseases, etc. [1,2,3,4]. In particular, the ratio of the lunula to the plate nail and the tiniest differences in nail colors can reflect ongoing human health status. Therefore, it is relevant to develop a first self-care health sensor method to detect nail color changes as an alternative to dermatological exams and scanning tests.

Advances in Artificial Intelligence (AI)—including fast Convolutional Neural Network (CNN) object detection models and machine learning—allow us to perform accurate tracking using only hand images taken from a HD webcam. Combining these advancements with libraries like TensorFlow, AI provides an efficient framework for deploying interactive algorithms for object detection and hand-tracking. With the use of sufficiently large datasets of diverse hand postures, CNNs can provide an alternative approach to train models that perform reasonably well when addressing challenges such as changes in lighting conditions, noisy environments, different hand viewpoints, and the account of finger occlusion when carrying out measurements.

Several prototypes have been proposed to detect fingernails and hand gesture interactions from computer vision [5,6]. One way is to develop a pre-trained hand detection model with CNNs and Deep Neural Networks. Different images in the given datasets, containing particular hands gestures as reference frames, allow to identify different input finger edges and areas around the finger plates. In training a hand detector sensor, multiple processes are needed to assemble a dataset, cleaning and splitting it into training/test/validation partitions and generating a display interface. This is built using data from unique hand datasets available on the Internet [7,8]. These datasets usually contain pixel-level annotations where hands are located across hundreds of high-quality images. Images are captured from egocentric and alternative views across different indoor and outdoor environments and activities using hand positions such as playing games and others. Hence, fast CNNs or deep learning algorithms with pre-trained models are an attractive candidate for real-time fingernail detection and tracking applications such as the one we report in this paper.

In this work, we introduce the NAILS system, a self-care health monitoring tool that uses an AI sensing method to analyze fingernail plates and color changes for early detection of health issues. Here, the NAILS stands for Normalized Artificial Intelligence Labeling Sensor. It leverages a pre-trained CNN model to segment and label fingernail regions from webcam images, normalizing RGB values to monitor in real time color changes that may indicate health concerns, such as oxygen deprivation or nutritional deficiencies. Particular snapshots of fingernails are used as reference frames, which allows us to identify different fingernail edges and nuances in real time. This sensor-based approach provides a non-invasive, real-time monitoring option through a user-friendly GUI, supporting primary self-care diagnostics rather quickly. The use of normalized RGB values combined with AI-based segmentation for real-time health monitoring is novel and innovative.

## 2. Previous Work

Some hand tracking and detection algorithms that use computer vision are not very robust, especially when these are based, for example, on extracting boundary features and backgrounds depending only on texture. This is also the case when distinguishing between hands and background using just histograms or color maps. In fact, on unusual backgrounds where varied or poor lighting causes changes in skin color or under situations in which the tracked hand becomes partially occluded, classical algorithms can easily lead to incorrect results.

There are a few more innovative and alternative AI-based, open-source software applications available which allow us to identify fingernails by applying CNN and image processing algorithms. One of these is nailtracking [6]. This is a real-time nail-detection algorithm using a CNN on TensorFlow. The deep learning framework or TensorFlow object detection allows to simplify the process of training a model for custom object detection. It allows, for instance, to draw a line when identifying the dynamics of a finger in action over a surface. The goal behind nailtracking is to provide an adaptable Python code to demonstrate how CNNs can be applied to tracking hands having egocentric and alternative views. The different hand images in the given datasets contain particular gestures which are used as reference frames and lead to the identification of different finger edges.

Another available algorithm for nail segmentation using deep learning models is denoted as nails_segmentation [5]. For the segmentation part, this algorithm employs the encoder–decoder structure DeepLabV3+ [9], which allows the fast recovery of the object boundaries. nails_segmentation consists of four basic steps for data preparation, training, prediction, and evaluation. For performance on diverse public datasets, this model can achieve higher than 80% success without the need of post-processing. Furthermore, if there is a pre-trained model (denoted “best_model.pth”), new results can be retrieved faster from scratch using that given model. For these two reasons, we adopt nails_segmentation as the AI-based algorithm to construct our GUI and easily identify the plate areas and colors of fingernails by image processing in real time, as discussed below. Normalizing RGB values to monitor and plot tiny color changes via a GUI and the use of an HD webcam in real time are the novel and innovative features of the present NAILS algorithm.

## 3. NAILS Algorithm

**Figure 1:**
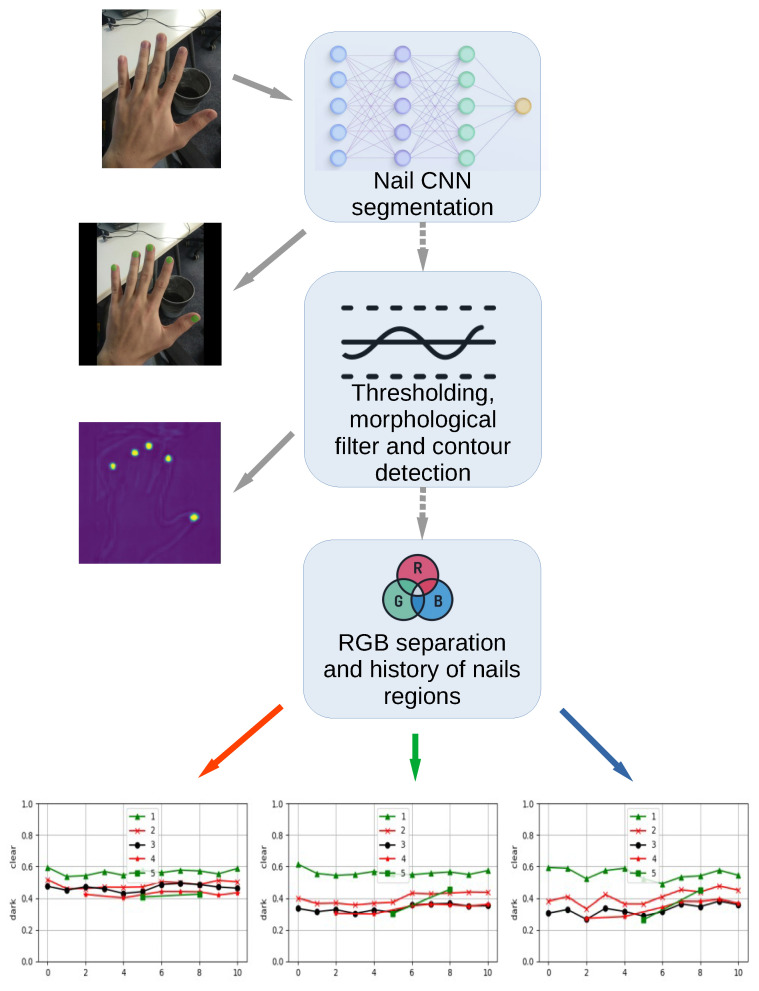
Diagram of the NAILS sequence algorithm. The hand images are examples from the dataset in [7].

The idea behind NAILS is to develop an algorithm aiming at detecting fingernail colors by image processing in real time by using the CNN nails_segmentation method [5]. We have found that this CNN performs better than alternative solutions because it is based on nail segmentation using fast deep learning models. Training is performed using public datasets comprising fingernail images of individuals. The dataset for nail segmentation, with images and annotated masks can be found in [7,8]. Usually, these masks are cleared up from the background noise, which may appear because these are created using gray and white color ranges. A median blur is used to fill holes in the masks. The weights are applied to real images with reasonably good results. It has to be noted that a properly trained CNN can effectively treat skin tones and lighting condition variations in a more robust way with respect to the classical segmentation approach. As described in Section 4, the lighting variations can be mitigated by enclosing the acquisition system inside a box, where the illumination is steady and controlled. We classify the images on the basis of fingernails implemented following the flow diagram in Figure 1.

**Figure 2:**
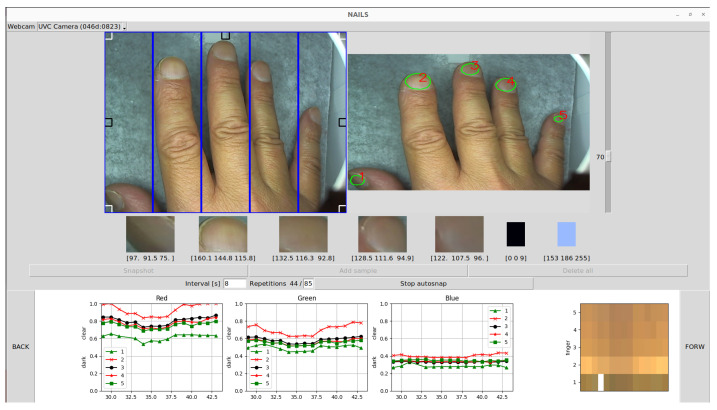
GUI for the Normalized Artificial Intelligence Labeling Sensor (NAILS) aiming to identify hidden signals from fingernails. The numbers 1-5 label each R-G-B curve with a corresponding fingernail.

When the user obtains a snapshot from the video source showing an open hand (subdivided in five equally rectangular areas separated by blue lines, as in the input video on the left of Figure 2), the NAILS system:

- Searches for and processes reference black and white regions in order to normalize the image pixels ($I_{x}$) and therefore the color values of the nails. The algorithm then normalizes the R(ed), G(reen), and B(lue) values at pixel *x* according to the equation:
  (1) $$\eta_{x}=\frac{I_{x}-Black_{x}}{White_{x}-Black_{x}}$$
  where *x* can be an R, G, or B region displayed on the left video.
- The referential black value is evaluated taking into account the minimum value inside the small-squared regions located at the four corners of the input video. The referential white value is evaluated taking into account the maximum value inside the small-squared regions located at the three central areas of the input video.
- Then, the CNN is fed by the acquired image in order to obtain the nail regions. The CNN uses the convolution layers (or layers that use specialized linear operations instead of the matrix multiplications) to extract features from the target objects. In our case, the CNN from [5] has been adapted to run under the Ubuntu O.S. This network performs better than alternative solutions because it is based on the segmentation concept. Segmentation is the process of dividing a digital image into different parts, in which each part consisting of homogeneous pixels distinguishes the object or other information contained in the image. The network has been trained over the default dataset and the trained weights are applied to real images with reasonably good results.
- The mask evaluated by the CNN network provides regions where nails are present.
- The mask is post-processed with threshold and morphological filters to improve the previous detection to obtain sharp contours around the nails.
- Finally, the system gets the RGB values from the nail regions (based on pixel values inside contours), normalizes the RGB values, and stores the RGB normalized values.
- As illustrated in Figure 2, the RGB normalized values of the respective R, G, B curves for each fingernail identified by the algorithm are plotted in the bottom region of the GUI, where the RGB history is displayed.

The minimum hardware needed to test the NAILS is any standard PC Computer, e.g., Intel Core i5, 64bit, and at least 4G RAM (ACER Aspire 5 or newer), running a recent release of the Linux O.S. (24.04 LTS or newer) and having an internal webcam and Python installed (deb python3 (3.10.6-1 22.04.1)).

**Figure 3:**
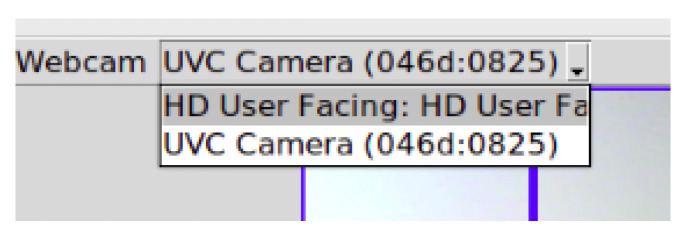
Webcam selection menu.

In the case of using an external USB webcam, the user needs to install the v4l-utils package, which can enumerate and detect many aspects of installed cameras. This class has been mainly added to the NAILS in order to implement the camera combo box to allow the user to change the current acquisition camera using the menu in Figure 3. Optional hardware that can be used is an external USB webcam (with optical zoom).

## 4. Fingernail Color Detection via NAILS

The GUI interface of the NAILS package shown in Figure 2 is based on the tkinter Python library (i.e., the standard Python interface to the Tcl/Tk GUI toolkit version 8.6). Some other libraries have been integrated in order to add plot history (with the matplotlib and numpy libraries being used) to show real time and the snapshots acquired from a USB webcam (opencv-python). Some classes have been developed in order to solve other tasks, such as videocapture pause and restart, complex processing of the acquired images, and so on. In order to mitigate the variations in the lighting conditions and to have a fixed background, the images can be acquired in a controlled environment. Even if the neural network is robust to the changes of external conditions, a closed box equipped with a controlled lighting system can provide a more repeatable and reliable environment.

In fact, the lighting conditions in the system can cause sharp changes in fingernail color. The partial occlusion of one tracked finger or situations when the fingernail area is too tiny to detect (e.g., half a thumb) can affect detection results, making it impossible to retrieve useful color data for that finger. For these reasons, it is advisable to insert the hand into a closed system (box) to make more precise measurements in a luminous controlled environment (e.g., resembling the ancient *“Mouth of Truth”* attraction in Rome, Italy). This allows to have every finger visible, illuminated and separated from the background.

To start using the NAILS GUI, the acquisition webcam from which to obtain the video image of the hand (input video on the left of Figure 2) needs to be first selected. The CameraSearch library was implemented in order to enumerate and to obtain information about the connected webcams: no one completely functional library was found from the open-source solutions available through the Internet. In order to make the CameraSearch library work, it is necessary to install the v4l-utils package, and hence our running GUI solution is currently not portable for Windows or MacOS systems. It can enumerate and detect many aspects of installed cameras. This class has been mainly used to implement the camera combo box to allow us to select the acquisition camera, as illustrated in Figure 3. In this figure, the menu listing one internal webcam and a second UVC webcam connected to the PC via USB is shown.

**Figure 4:**
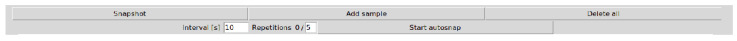
Modalities to acquire (single or multiple) images for the fingernail regions.

After positioning each finger in one of the five equally rectangular areas separated by blue lines in the GUI input video—used as guidance—there are two modalities to obtain the nail regions through the NAILS CNN method. As illustrated in Figure 4, one modality is to acquire single images for the nail regions by selecting and pressing the “Snapshot” button or just pressing the “Enter key”. When selecting the “Snapshot” single button in the GUI, one can “add sample” or press the “Space key” to plot the estimated single RGB values. After a few seconds, the identified areas around the fingernails are displayed (output image on the right of Figure 2). The second modality is to acquire a large temporal set of data at given intervals by selecting “Start autosnap”.

**Figure 5:**
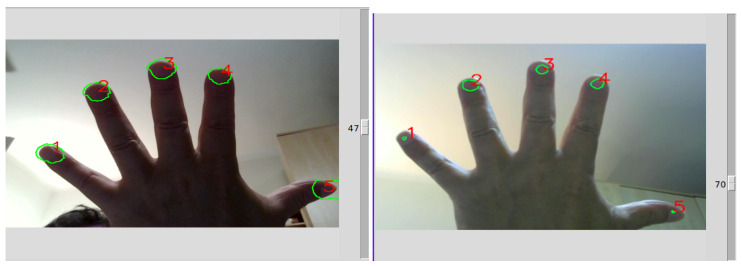
Fingernail areas regulated in size with the cursor on the right of the GUI.

The areas around the fingernails can be regulated in size with the cursor appearing on the right of the GUI as shown in the example in Figure 5. In all cases, the plotted values surrounding these areas correspond to a mean value obtained for each single fingernail region identified. By selecting “Delete all”, all recorded data are canceled.

**Figure 6:**
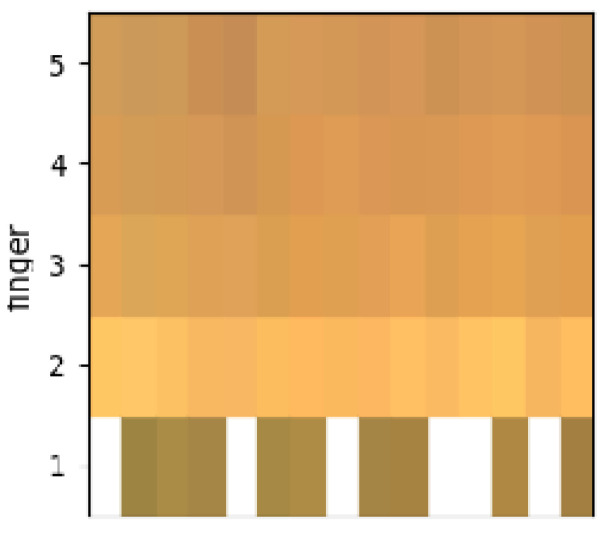
The visible RGB colors of the five fingernail regions being identified at each measurement. The empty white values correspond to missing measures with the CNN algorithm, usually due to the occlusion and position of the thumb with time. The observed nail color range is similar to the skin color and depends on luminosity.

The NAILS algorithm then obtains all the actual RGB colors of the (five) fingernail regions, as shown in Figure 6. These single colors are based on the theory that all visible colors can be recreated using the primary additive colors of red, green, and blue, assigning a value in the range of 0–255. When these values are combined in different amounts, fingernail colors can be produced. For example, (0, 0, 0) is black and (255, 255, 255) is white.

**Figure 7:**
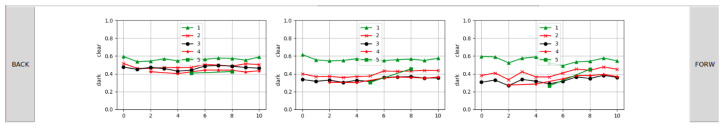
Red, green and blue plots of the mean color values for each fingernail.

As can be seen in Figure 7, the GUI shows in real time the three normalized R-G-B values identified by the algorithm for each fingernail region, normalized using Equation (1). The R-G-B recorded values during a period of time (configured at given intervals and repetitions within the GUI) can be visually compared by using the Forward and Backwards buttons on both edges of the plot.

## 5. Discussion

Our project aims at detecting symptoms of various diseases in their early stages. The NAILS may help to associate pre-symptoms according to the literature [1,10,11] since the color of fingernails may reflect the health status of an individual as, for example, those depicted in Table 1.

There are a few issues leading to noticeable performance increases in the results observed through the AI-based NAILS algorithm, and the CNN advances to make these results faster [12]. The CNN used is a type of neural network architecture with many sub-sampling layers capable of performing object detection with high-level accuracy [13]. Each layer in this network is able to extract the target object’s features. Thus, the NAILS can be used for real-time processing. The time in which the NAILS algorithm is able to process a measurement is less than 2 s (without GPU cores).

The size of the reading images from the webcam is set by default to 800 × 800 px to avoid slowing down the program in real time. Keeping these input images small allows us to increase the video fps rate without losing significant accuracy. The observations are made fast because a pre-trained model (namely, “best_model.pth”) is given with the sources of the NAILS, which is trained on the Ubuntu O.S. It took around two hours to generate the NN on an i9 CPU machine.

**Figure 8:**
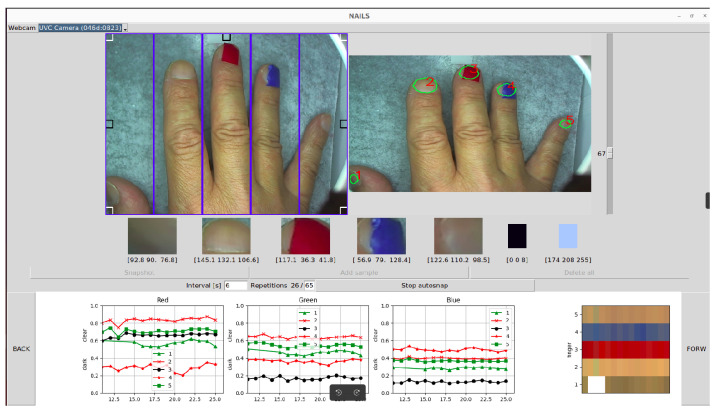
Critical assessment of NAILS behavior by forcing red and blue fingernail colors.

The NAILS does not deal with the segmentation of specific parts of the nail like the lunula. It uses a simple image processing method to segment nails from given images. The NAILS identifies and decouples visible RGB colors of fingernail regions at each measurement, as shown in Figure 6 and Figure 7. An example for a critical assessment of NAILS behavior (e.g., when forcing red and blue fingernail colors to picture effective changes) is displayed in Figure 8.

Let us construct next a quantitative framework to evaluate the performance of the proposed NAILS method. We use Intersection over Union (IoU) for object detection and segmentation in order to evaluate the segmentation quality against the Ground Truth (GT) nail masks derived through the NAILS algorithm. We also evaluate two different prior studies in the field [5,14] in terms of IOU for comparison with the NAILS. The results obtained are listed in Table 2.

To obtain these results in terms of Mean IoU Score and Mean Dice Loss, we have created new GT datasets by acquiring images via the NAILS interface properly adapted to process the generated images and their corresponding masks in real time. We then tested the CNN following [5] with these new datasets. In particular, we processed two datasets with different morphological cv2.erode filters to clean up the mask obtained from the NN and to eliminate possible connections between the identified regions of the fingernails. This allows us to verify on the GUI (see Figure 5) that the selected region circumscribes a nail or not, avoiding overlapping between fingernails. In this way, the contour calculation is cleaner and works better. The first NAILS dataset considered with erode_filter=70 contained 60 different images, and the second dataset with erode_filter=92 contained 50 images.

From the outcomes in Table 2, we found that the NAILS values with erode_filter=70 are essentially similar to those obtained with the test data provided from [5]. In both cases, the IoU is good and close to 1 and the Dice coefficient for segmentation approaches 0. The fingernail RGB color is evaluated in real time with the output shown in Figure 7. On the other hand, the IoU value with the dataset from [14] is not so good, mainly because this dataset is generic and the hand poses vary very much. In our system, the hand position remains fixed, so the NN can detect fingernails more easily with respect to a generic configuration. A difference with [5] is that the NAILS does not deal with the segmentation of the inner nails’ lunulae.

The proposed NAIL system is a combined system using [5], as can be seen from the similar results obtained with the datasets for the overlap of the GT and prediction region. It is expected that an alternative CNN for nail segmentation or improvements in the training dataset can be proposed in the future to enhance even further the performance of the system and to increase the accuracy and precision in detecting signs of disease.

According to the present results, our human fingernail segmentation with model training algorithm may then be used as the first self-diagnostic tool. The NAILS algorithm overcomes the limitations of human eye resolution and provides an objective evaluation of nail color. In all cases, however, a definitive diagnosis is always up to a dermatologist specialist since laboratory tests are always necessary. Hidden signals in the discoloration of the nails using AI, together with the observation of other symptoms and physical examination, could be useful for diagnosing a specific disease, especially in remote areas or when standard testing by accurate reading machines is not being used or is not always available.

## 6. Conclusions

We have introduced NAILS, an AI-based, non-invasive, sensor-like method for inspection of fingernails through segmentation and labeling. We built a generalized model to identify single fingers, plates, and color data. In the construction, a pre-trained deep learning neural network containing different images of fingernails has been used as the reference frames to identify fingernail edges and color areas in real time. The RGB average value of the input fingernail color can be used to classify some diseases. A first assessment of the accuracy of the NAILS was performed via IoU.

The main aspect of the NAILS algorithm is that it allows to analyze—via a simple GUI and a USB webcam—the tiniest dynamic changes in fingernail colors. This feature could be used as an indicator of human health status. Generally, healthy fingernails are shiny and smooth in appearance. From the algorithmic point of view, further developments can be implemented by modifying the current CNN with new training sets and by considering more recent methods for segmentation, like in Ref. [15]. Even the normalization step can be more deeply investigated in order to improve the robustness of the color classification. A Retinex approach could be applied to develop this aspect.

We believe the NAILS opens the door to run on different configurations. For example, integrating multi-sensor data to control the level of light uniformity inside the NAILS box system, as in [16], may help to enhance higher diagnostic accuracy. In addition, the NAILS algorithm could also be extended to explore applications on mobile devices to identify potentially life-threatening illnesses in real time, such as the app discussed in Ref. [17] to detect levels of anemia characterized by low blood hemoglobin (Hgb) levels. By scanning fingernails’ colors, the NAILS could also be expanded to detect a broader range of symptoms than those listed in Table 1. For instance, it could be adapted to detect the presence of remaining particles of nail polish (gel), which impact the accuracy of pulse oximeter readings [18]. The NAILS algorithm can also be embedded in a Raspberry Pi with Alpine Linux installed. All of these directions have the potential to be explored in future releases.

## Figures and Tables

**Table 1:** Nail color and disease symptoms.

Nail Color	(RGB)	Examples of Possible Diseases
Yellow	(255, 255, 0)	Diabetes or psoriasis, lung disease
Green	(0, 255, 0)	Allergies to cleaning agents, localized fungal infection
Brown	(165, 42, 42)	Arsenic or copper poisoning, nicotine
Red	(255, 0, 0)	Injury, splinter hemorrhage, high blood pressure
White	(255, 255, 255)	Protein deficiency, anemia
Purple	(128, 0, 128)	Oxygen deprivation, circulatory problems

**Table 2:** IoU evaluation of different test data.

Values	Test Dataset from [5]	Roboflow Dataset [14]	NAIL Dataset (Filter 70)	NAIL Dataset (Filter 92)
Mean IoU Score:	0.9571	0.5673	0.9439	0.9001
Mean Dice Loss:	0.2565	0.4764	0.2978	0.3122

## Data Availability

Code, manuals, datasets analyzed and generated during the study can be found at https://github.com/canessae/nails (accessed on 8 December 2024).
